# New onset epilepsy in the elderly: clinical, radiological and electroencephalographic features and treatment responses

**DOI:** 10.17712/nsj.2017.2.20160527

**Published:** 2017-04

**Authors:** Erum M. Shariff, Fahd A. AlKhamis

**Affiliations:** *From the Department of Neurology (Shariff), King Fahd Medical City, Riyadh, and from King Fahad Hospital of the University (Shariff, AlKhamis), University of Dammam, Al-Khobar, Kingdom of Saudi Arabia*

## Abstract

**Objective:**

To evaluate new onset epilepsy characteristics, etiology, radiological and electroencephalographic features and to document treatment response in the elderly.

**Method:**

This was a retrospective study carried out in King Fahd Medical City, Riyadh, Kingdom of Saudi Arabia, from 2010 to 2013. Medical records were searched to recruit patients. Hundred and nineteen patients were enrolled who fulfill the inclusion criteria. Clinical data with respect to seizure semiology, etiology, electroencephalographic findings, radiological findings, co-morbidities, and anti-epileptic drug (AED) therapy were assessed.

**Results:**

Cerebrovascular disease was the most common etiology, followed by occult cerebrovascular disease (oCVD). Focal onset seizures were the most common clinical presentation in this group of cohort. Electroencephalogram (EEG) showed generalized slowing in majority of patients (45.5%), with 21.8% interictal epileptiform activity. Patients required lower doses of AEDs to control seizures, gain better seizure control. Common co-morbidities were hypertension and diabetes mellitus.

**Conclusion:**

Patients presenting with LOE, should have search for any other cerebrovascular risk factors. Further research is needed to determine the prevalence of oCVD in LOE, and to investigate whether addressing cerebrovascular risk factors in this cohort of patients can reduce the incidence of stroke.

The prevalence and incidence of epilepsies in elderly is high, a third of all diagnoses of epilepsy are made in people over 60.^1^,^2^ Due to demographic development, the portion of elderly patients with epilepsy will continue to rise over the next decades. In some industrialized countries, one third of the population is expected to be over the age of 65 in 2030.^3^ Epidemiologic studies have shown that the incidence of epilepsy is significantly higher in the elderly than in any other age group.^4^-^9^ One study found that the incidence of epilepsy occurring at the age of 70 years old is almost double that of children, and by the age of 80 years old, more than 3 times the incidence during childhood.^10^,^11^ Therefore, the number of elderly patients suffering from epilepsy will be high, and most of them will likely have other co-morbidities. This increase will put a heavy burden on health care and pension systems. The rate of mortality resulting from epilepsy increases when epilepsy is coupled with cardiovascular diseases or senile dementia.^3^,^12^-^13^ Diagnosis of epilepsy in elderly can be difficult and may require long-term electroencephalogram (EEG) monitoring, moreover; it has specific features, including different aspects of etiology, clinical manifestations, and treatment responses, which are different from epilepsy in younger individuals. Clinicians who treat epilepsy in the elderly should be aware of these important characteristics. Epilepsy in elderly is a diagnostic challenge that is impeded by several factors: (1) patient often cannot provide a detailed history of their seizures, and these seizures are often not observed by other. (2) Often diagnosed as altered mental status, memory lapses, confusion, transient ischemic attack (TIA), or late life migraine accompaniments.^3^,^14^ The objective of this study is to identify the characteristics and etiological diagnosis of epilepsy in the elderly in Saudi Arabia, we reviewed our experience at a tertiary care referral center.

## Methods

This was a retrospective study. Patients were selected from the hospital records of the epilepsy patients in King Fahd Medical City, Riyadh, Kingdom of Saudi Arabia. After getting institutional review board (IRB) approval, we searched all electronic medical records to identify cases of new-onset epilepsy in elderly. Patient enrolment began on August 2013, and patients presented between 2010 to 2012, who fulfilled the criteria were recruited. We defined elderly population as age 60 years and above. Seizures were classified according to the revised International League against Epilepsy classification into primary generalized, simple partial seizure complex partial seizures and secondary generalized.^15^ All the patients were enrolled who fulfill the inclusion criteria. Inclusion criteria: All the patients above 60 years of age, both male and female, who presented with new onset seizure, or their first seizure was at age equal or above 60 years were enrolled. Exclusion criteria: Patients with overt sepsis, hepatic encephalopathy, alcohol withdrawal seizures, and gross metabolic abnormalities; which likely to provoke seizures, namely (i.e.) hypoglycemia, hyperglycemia (serum level <60 mg/dl, or >600 mg/dl), hypocalcaemia (serum level <2.2 mmol/lit), or hyponatremia (serum level <125 mg/dl) were excluded. Clinical, radiological, and EEG findings, AED doses, and treatment responses were recorded in a pre-formed Performa. Statistical analysis was carried out using Statistical Package for the Social Science Version 20 (SPSS Inc., Chicago, IL, USA). This study was conducted according to principles of Helsinki Declaration.

## Results

**Figure 1:**
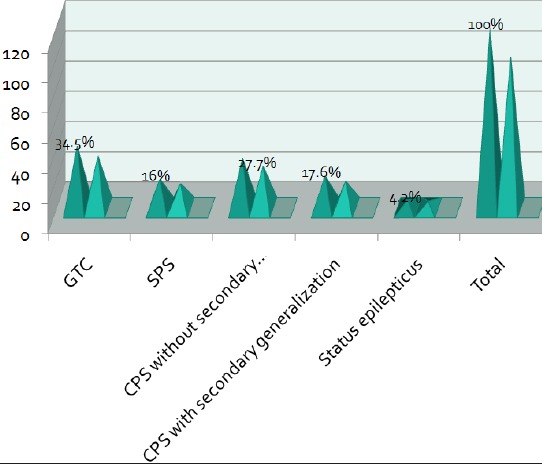
- Seizure Classification. CPS - complex partial seizure, SPS - simple partial seizure, GTC - generalized tonic clonic seizure

**Figure 2:**
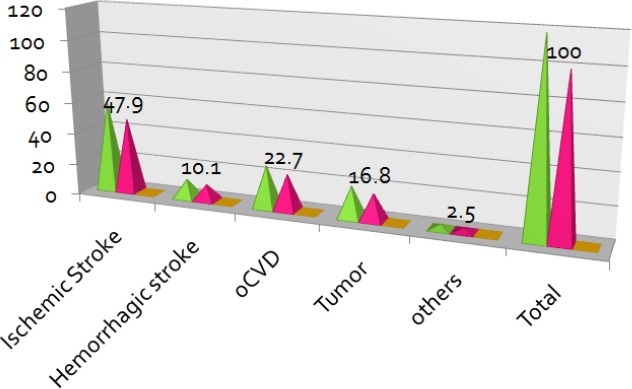
- Etiology of seizure. oCVD - occult cerebrovascular disease

Hundred and nineteen patients were enrolled in the study; their mean age was 70.12±8.72 years (mean±SD) and 59 (49.6%) were male. According to seizure classification 73 (61.3%) patients presented with focal onset; with or without secondary generalization, 33 (27.7%) patients with complex partial seizures (CPS) without secondary generalization, 21 (17.6%) CPS with secondary generalization and 19 (16%) with simple partial seizure (SPS), followed by generalized tonic clonic seizure (GTCs) in 41 (34.5%) and status epilepticus in 5 (4.2%) (**Figure 1**) An etiological diagnosis was possible in 92 (77.3%) of patients, including those with cerebrovascular disease such as ischemic or hemorrhagic 69 (58%), followed by tumors 20 (16.8%) and other etiology including infection, trauma and other inflammatory conditions in 3 (2.5%) patients (**Figure 2**). Around 27 (22.7%) patients who were labeled as cryptogenic or non-lesional found to have oCVD in the form of small vessel disease, brain atrophy, and subcortical infarcts. Interictal EEG showed; focal epileptiform discharges in 26 patients (21.8%), while slowing in 53 patients (44.5%). Other finding including periodic lateralized epileptiform discharges (PLEDS), and triphasic waves in 7 patients (6.2%), normal record in 20 patients (15.9%) while EEG was not available in 13 patients (8.8%). Seven patients presented with status epilepticus, 5 out of them had ischemic stroke, while one had tumor and one found to have oCVD. Of the 119 patients 107 were on monotherapy and 6 (5%) were on 2 drugs, and 5% were on no medications (**Table 1**).

**Table 1:** Number and frequency of antiepileptic medications.

Medications	n	(%)
Levetiracetam	60	(50.4)
Phenytoin	37	(31.1)
Valproic acid	6	(5.0)
Carbamazepine	4	(3.4)
No treatment	6	(5.0)
Polytherapy	6	(5.0)

## Discussion

As the elderly population is growing in the Kingdom of Saudi Arabia, an increased incidence of stroke is also expected.^10^ Stroke is the leading cause of epilepsy worldwide and can be associated with both immediate and delayed epileptic seizures, leading to an increased risk of epilepsy 5.7% within first year and 11.5% within 5 years.^16^ It has been reported that the most common cause of seizures in LOE is cerebrovascular disease; other etiologies include trauma, degenerative diseases, congenital malformations, brain tumors, or CNS infections.^9^,^17^-^19^ Our findings are consistent with previous studies of stroke as the underlying etiology in more than half of the patients. Stroke was the etiology in 69 patients (58%); brain tumor was found in 20 patients (16.8%), other etiologies including infection and trauma was found in 3 patients (2.5%). The oCVD was found in 22.7% of patients, who were labeled as cryptogenic at the time of their first seizure. This was the second most common etiology in our cohort of patients. Epileptic seizures and cerebrovascular disease show a bidirectional relation, there is increasing evidence that even oCVD is associated with increased risk of developing epilepsy in this population.^20^,^21^ The possible mechanisms of epileptogenesis in otherwise oCVD may include disruption of neurovascular unit integrity; disordered cerebral metabolism and perfusion; blood-brain barrier dysfunction; and inflammation.^22^ The oCVD may be detected on brain imaging but by definition does not manifest otherwise clinically. Structural imaging markers of oCVD are thought to include cortical or subcortical infarcts, white matter hyperintensities, leukoariosis, cerebral atrophy, and brain micro-bleeds which are marker of cerebral microangiopathy, and strongly associated with HTN.^22^

Recent studies have also found this increased stroke risk not to be limited to elderly but present in patients diagnosed with epilepsy as young as 20 years of age.^23^,^24^ One study demonstrated patients with epilepsy after the age of 40 years; had significantly more oCVD, compared to their age-matched controls. While 12 of the 56 patients had their first seizure after the age of 60 years, had infarcts on CT scan compared with one of the 57 controls, giving a prevalence of oCVD in patients with LOE of 21.4%.^25^,^26^ This increased risk of stroke in patients with LOE should dictate the evaluation and management of stroke risk factors to prevent stroke. Our results showed a higher rate of CVD compare to other recent studies, our study conducted at a tertiary care referral hospital; therefore; it may have been affected by significant referral biases.

In the current study focal onset seizures were the most common seizure type (61.3%). These findings are in accordance with previous studies reporting that the most frequent type of seizures in the elderly is CPS.^3^,^4^,^9^,^27^ One study from Kingdom of Saudi Arabia showed 53% had focal onset while 24% had generalized onset seizures, unclassified in 17.5%; and status epilepticus in 5.3% of patients.^10^ Elderly presenting with CPS are often misdiagnosed as having altered mental status, memory disorders, or episodes of confusion. The lack of secondary generalization makes these seizures more difficult to recognize and classify, which may often lead to difficulty in diagnosing epilepsy. Stroke mimic was the final diagnosis in one recent series of 350 clinical presentations of suspected stroke, representing the 31% of cases. Seizures accounted for 21% of all stroke mimics and 29% of stroke mimics presented within 6 hours.^25^ We had 5 patients with status epilepticus, 4 out of those had cerebrovascular disease. One study showed that status epilepticus was more frequent in older patients than in younger adults.^3^

The IEA in our study was found in 21.8% of patients, while a large number of patients had non-specific findings of generalized or lateralized slowing (46%). Results are controversial; one recent study showed that 72.9% patients had IEA^9^ while a previous study showed that IEA was present in 26 % patients with seizure onset after 60 years.^28^ Therefore, the variability in the rate of IEA in routine EEG studies must be considered when making the diagnosis of epilepsy syndrome for episodic events occurring in the elderly. The HTN was the most prevalent (57%) comorbidity found in our study followed by DM (55%). This is in line with the previous reports.^10^ The relationship between hypertension and seizures through brain damage with or without manifest stroke has been investigated and found that hypertension significantly associated with unprovoked seizures.^25^,^29^,^30^ Most patients with newly diagnosed epilepsy responded to treatment with their first AED. In fact, 89.9% of our patient population was seizure free on one medication. Previous studies have shown that seizures in elderly patients respond well to treatment and that AEDs effectively control seizures in approximately 77-86%.^9^,^31^,^32^ Most AEDs are effective for treating common seizures in the elderly.^33^ Levetiracetam was the most commonly used AED in our patients cohort. Accurate classification of seizures is very important to ensure an appropriate choice of AED. The elderly patients in our study became seizure-free after receiving a relatively low dose of medication, which is consistent with the results of previous reports.^9^

A limitation of this study may be that only patients from a tertiary care hospital were recruited, that could account for higher percentage of co-morbid conditions. Relationship between stroke and LOE is very interesting; stroke is the most common etiology in LOE while LOE posses an increase risk of subsequent stroke.^23^,^24^ In order to get better awareness of the disease and understanding the diagnostic challenges of LOE; there is tremendous need to find out the prevalence and the actual burden of the disease.

In conclusion, most common seizure type in our study was focal onset, stroke was the common cause of late onset epilepsy, and even oCVD was prevalent. The HTN and DM were commonly associated co-morbidities. Patients presenting with LOE, should have search for any other cerebrovascular risk factors. Given the small sample size, further research is needed to determine the prevalence of occult CVD in LOE, and to investigate whether addressing cerebrovascular risk factors in this cohort of patients can reduce the incidence of stroke. A prospective study will be of great value in the future.
